# Proteolytic enzymes from *Bacillus subtilis* AB2 as antibiofilm adjuvants: Bioprocess optimization, mechanistic insights, and synergy with antibiotics

**DOI:** 10.71150/jm.2509019

**Published:** 2025-12-31

**Authors:** Afra M. Baghdadi

**Affiliations:** Department of Biological Sciences, College of Sciences, University of Jeddah, Jeddah 21589, Saudi Arabia

**Keywords:** collagenase, keratinase, *Bacillus subtilis*, biofilm, diabetic foot infection, enzymatic debridement, wound adjunct therapy

## Abstract

Collagenase and keratinase are two important proteolytic enzymes with recognized applications in biotechnology and medicine, particularly in the enzymatic removal of necrotic tissue and the control of infection. In the present work, a soil isolate of *Bacillus subtilis* strain AB2 (PX453297.1) was optimized for enzyme production under different nutritional and physicochemical conditions. The enzymes were recovered by ammonium sulphate precipitation and dialysis, examined by SDS-PAGE and zymography, and further assessed for pH and temperature optima, stability, the influence of metal ions, and kinetic parameters. Maximum collagenase activity (4.41 ± 0.22 U/ml) was observed at 37°C and pH 7.5 in a glucose–peptone medium, whereas keratinase production was enhanced between 37 and 40°C at pH 7.5 in lactose–peptone medium. Protein bands of approximately 55 and 33 kDa were detected, representing 6.2- and 5.5-fold purification. Collagenase showed an alkaline optimum (pH 10.0, 37–45°C) with K_m_ 0.31% and V_max_ 1.92 U/ml, while keratinase exhibited dual optima (pH 3.0 and ~7.0) with K_m_ 0.27% and V_max_ 0.84 U/ml. Biofilm assays revealed that collagenase reduced pre-formed biomass by 62–68% and viable counts by 1.1–1.7 log_10_, clearly outperforming keratinase (41–57%, 0.7–1.2 log_10_). When combined with conventional antibiotics, both enzymes potentiated activity, with notable synergy between collagenase and oxacillin against *Staphylococcus aureus* (FICI 0.31–0.37), ciprofloxacin against *Pseudomonas aeruginosa* (FICI 0.37–0.50), and meropenem against *Klebsiella pneumoniae* (FICI 0.28–0.44). These results indicate that *B. subtilis* AB2 produces collagenase and keratinase with distinct biochemical characteristics and strong antibiofilm properties, underscoring their promise as adjuncts in chronic wound care as well as in industrial applications.

## Introduction

Proteolytic enzymes, particularly collagenases and keratinases, have attracted significant attention due to their diverse industrial and biomedical applications. Collagenases, which degrade collagen as the major structural protein of connective tissues, are widely applied in biomaterials processing, pharmaceutical formulations, and enzymatic debridement of chronic wounds. Keratinases, on the other hand, have the unique ability to hydrolyze keratin, an insoluble and highly stable protein present in feathers, hair, nails, and stratum corneum, making them valuable for the valorization of keratinous wastes, textile processing, and potential therapeutic uses in skin and wound care (Li, 2021).

Compared to plant or animal-derived enzymes, microbial proteases are particularly attractive because of their scalability, tunable properties, and the feasibility of tailoring them for use in complex substrates and physiological conditions. Recent reports have highlighted soil-derived *Bacillus* species as prolific producers of keratinases and collagenases with promising stability profiles and bioprocessing potential, such as *Bacillus paralicheniformis* T7 which demonstrated high activity and strong thermal tolerance in feather-waste degradation (Aktayeva and Khassenov, 2024). Similarly, *Bacillus cereus* keratinase has been shown to enhance transdermal antibiotic delivery by facilitating matrix penetration in infection models, underscoring the relevance of these enzymes in biomedicine (Sypka et al., 2021).

The clinical relevance of proteolytic enzymes has been further reinforced in the context of chronic wounds, including diabetic foot ulcers, which remain a major health burden worldwide. One of the key reasons for poor wound healing outcomes is the persistence of bacterial biofilms, which are structured microbial communities encased in an extracellular polymeric substance that protects pathogens from immune responses and antibiotic therapy (Diban et al., 2023). Biofilm formation by common wound pathogens such as *Staphylococcus aureus*, *Pseudomonas aeruginosa*, and *Klebsiella pneumoniae* complicates treatment and is a major driver of infection chronicity. Enzymes capable of degrading proteinaceous or keratinous components of the biofilm matrix have emerged as promising adjunct therapies, as they can weaken biofilm architecture, facilitate antibiotic penetration, and enhance wound healing outcomes (Al-Madboly et al., 2024). Despite these promising leads, most studies still focus on the discovery of either keratinase or collagenase individually, often emphasizing waste valorization or industrial applications without fully addressing their translational potential in biomedical contexts.

Another important gap in the current literature is the lack of systematic and comparative characterization of both enzymes from the same microbial strain under identical production conditions. While a few studies report on production optimization for either keratinase or collagenase, detailed assessments of environmental parameters such as temperature, pH, inoculum density, substrate load, and carbon or nitrogen sources are often partial and rarely analyzed statistically (Aktayeva and Khassenov, 2024). Similarly, purification efforts are frequently limited to crude extracts, with few reports providing purification folds, yields, and SDS-PAGE validation. Moreover, the biochemical characterization of these enzymes-such as kinetic parameters, stability across pH and temperature ranges, and sensitivity to metals or inhibitors-remains incomplete in many works, reducing their translational value.

In light of these gaps, we investigated *Bacillus subtilis* AB2, an isolate obtained from arid soil in the Makkah region of Saudi Arabia, as a dual producer of collagenase and keratinase. The present study sought to optimize production conditions for both enzymes, purify them to homogeneity, and determine their biochemical properties including kinetic constants, stability profiles, and responses to inhibitors and metal ions. Beyond enzymology, we aimed to evaluate their translational potential by quantifying their capacity to disrupt pre-formed biofilms of *S. aureus*, *P. aeruginosa*, and *K. pneumoniae* and assessing their ability to potentiate frontline antibiotics in vitro. By combining bioprocess optimization, biochemical characterization, and biofilm-targeted applications, this work provides a comprehensive assessment of collagenase and keratinase from *B. subtilis* AB2 and highlights their potential as enzymatic adjuvants in the management of biofilm-associated infections such as diabetic foot ulcers (Cavallo et al., 2024). *Bacillus subtilis* was specifically selected for this study because of its long-established safety (Generally Recognized as Safe, GRAS) status, genetic stability, and exceptional ability to secrete large quantities of extracellular enzymes, particularly proteases with high catalytic efficiency and industrial relevance (Gupta et al., 2002; Rai and Mukherjee, 2010). The species exhibits remarkable metabolic versatility, enabling growth on a wide range of substrates, including collagen and keratin, while maintaining enzyme activity across broad pH and temperature ranges (Cai et al., 2008; Majeed et al., 2024). In addition, *B. subtilis* proteases are known for their thermostability, tolerance to metal ions, and resistance to denaturation-features that enhance their potential for biomedical applications such as wound debridement and biofilm disruption (Beg and Gupta, 2003; Leistikow et al., 2024). Accordingly, the isolation of strain AB2 from arid soil in the Makkah region provided a promising candidate for the dual production of collagenase and keratinase under optimized conditions for prospective therapeutic and biotechnological use.

## Materials and Methods

### Microorganism and culture conditions

The bacterial isolate designated AB2 was recovered from soil samples collected at Huda al-Sham, an arid agricultural region in the Makkah province, Kingdom of Saudi Arabia. Soil samples were aseptically collected at a depth of 5–15 cm, transported to the laboratory in sterile polyethylene bags, and processed within 24 h. Serial dilutions were plated on nutrient agar and incubated at 30°C for 24–48 h to recover bacterial colonies with distinct morphologies. Colonies with proteolytic halos on skim-milk agar were selected for further screening, and isolate AB2 consistently showed strong gelatin and feather-meal hydrolysis.

For maintenance, AB2 was cultured on nutrient agar slants and stored at 4°C for short-term use, with glycerol stocks (20% v/v) preserved at –80°C for long-term storage. Working inocula were prepared by cultivating the strain in nutrient broth at 30°C, 120 rpm for 18–24 h, and these precultures were used to seed production media for collagenase and keratinase assays.

### Molecular identification

Genomic DNA of isolate AB2 was extracted from overnight cultures using the phenol–chloroform method as described by Sambrook and Russell (2001). The nearly full-length 16S rRNA gene was amplified by PCR using the universal bacterial primers 27F (5′-AGAGTTTGATCMTGGCTCAG-3′) and 1492R (5′-TACGGYTACCTTGTTACGACTT-3′), which were originally reported by Weisburg et al. (1991). Amplification reactions were carried out in 50 µl volumes containing 25 µl of 2× PCR Master Mix (Thermo Fisher Scientific, USA), 1 µl of each primer (10 µM), 2 µl of genomic DNA (~50 ng), and nuclease-free water, under the following conditions: initial denaturation at 95°C for 5 min; 35 cycles of denaturation at 95°C for 30 s, annealing at 55°C for 30 s, and extension at 72°C for 90 s; followed by a final extension at 72°C for 10 min. The expected ~1,462 bp amplicon was confirmed by agarose gel electrophoresis (1.2% w/v) and subsequently purified using a QIAquick PCR purification kit (Qiagen, Germany). Bidirectional sequencing was performed with Sanger chemistry on an Applied Biosystems 3730XL DNA Analyzer, and the consensus sequence was assembled and analyzed against the NCBI GenBank database using BLASTn (PX453297.1). The isolate showed ≥ 99.3% sequence similarity with reference *Bacillus subtilis* strains, supporting its assignment at the species level. For phylogenetic analysis, the AB2 16S rRNA sequence and closely related reference sequences were aligned with ClustalW in MEGA X (Kumar et al., 2018), and a Neighbor-Joining tree was constructed with 1,000 bootstrap replications, placing AB2 firmly within the *B. subtilis* clade with strong bootstrap support values (> 90%). To complement molecular characterization, biochemical profiling was performed using the API 50 CHB system (bioMérieux, France), which confirmed carbohydrate utilization patterns consistent with *B. subtilis*.

### Media and production

For enzyme production, *B. subtilis* AB2 was cultivated in defined media optimized for collagenase and keratinase synthesis. Collagenase production medium was prepared according to a modified mineral-salts formulation (Tran and Nagano, 2002), containing (per L): KH_2_PO_4_, 2.0 g; K_2_HPO_4_, 7.0 g; MgSO_4_·7H_2_O, 0.1 g; citric acid, 0.05 g; yeast extract, 1.0 g; CaCl_2_·2H_2_O, 0.1 g; and gelatin, 3.0 g. Gelatin served both as a nitrogen source and an inducer of collagenase synthesis. For activity assays, bovine type I collagen (Sigma-Aldrich, USA) was supplemented as the substrate to quantify enzymatic hydrolysis.

Keratinase production medium was based on the feather-meal basal medium originally described by Agrahari and Wadhwa (2010). Each liter contained: chicken feather powder, 10.0 g (washed, dried, and ground to < 1 mm particle size); KH_2_PO_4_, 0.4 g; K_2_HPO_4_, 0.3 g; and NaCl, 0.5 g. The pH was adjusted to 7.5 prior to sterilization. Feather powder was sterilized separately by autoclaving at 121°C, 15 psi for 20 min, and added aseptically to the basal salts to minimize nutrient caramelization.

Both media were distributed as 50 ml aliquots in 250 ml Erlenmeyer flasks, providing a 1:5 liquid-to-air ratio to ensure adequate aeration. Media were sterilized by autoclaving at 121°C, 15 psi for 15 min. Flasks were inoculated with 2% (v/v) actively growing preculture of AB2, prepared by incubating the strain in nutrient broth for 18 h at 30°C, 120 rpm. Production cultures were incubated at 30°C with agitation at 120 rpm for 5–7 days.

Growth monitoring was performed by measuring optical density at 550 nm (OD_550_) using a UV–VIS spectrophotometer (LaboMed UV-2602, USA). Samples (2 ml) were withdrawn aseptically every 24 h, and cells were harvested by centrifugation at 10,000 × g for 10 min at 4°C. The cell-free supernatants were used as crude enzyme extracts for subsequent activity assays.

### Screening and enzyme assays

Collagenase activity was determined using insoluble collagen as substrate, following a modified ninhydrin-based spectrophotometric method (Kate and Pethe, 2022; Tran and Nagano, 2002). Briefly, 10 mg of insoluble bovine type I collagen (Sigma-Aldrich, USA) was suspended in 0.8 ml of 50 mM Tris–HCl buffer (pH 7.5) containing 4 mM CaCl_2_ and incubated at 30°C for 10 min with constant shaking (120 rpm) to equilibrate the substrate. The reaction was initiated by adding 0.2 ml of crude enzyme extract and allowed to proceed at 30°C for 30 min with agitation to maintain suspension of the collagen fibers. The reaction was terminated by adding 1 ml of 0.1 M acetic acid, followed by centrifugation at 10,000 × g for 15 min at 4°C to remove undigested collagen. The supernatant was analyzed using the modified ninhydrin method to quantify liberated free amino groups. Absorbance was measured at 570 nm (UV–VIS spectrophotometer, LaboMed UV-2602), and enzyme activity was expressed as micromoles of leucine equivalents released per min per ml of culture filtrate (U/ml). A negative control was prepared identically but without substrate addition to correct for background absorbance.

Keratinase activity was assayed according to a modified Lowry–Folin procedure (Agrahari and Wadhwa, 2010; Riffel and Brandelli, 2006). The reaction mixture consisted of 1.0 ml of cell-free supernatant and 10 mg of chicken keratin powder suspended in 1.0 ml of 50 mM citrate buffer (pH 5.0). Samples were incubated at 50°C in a water bath for 60 min with gentle shaking. The reaction was stopped by adding 2.0 ml of 10% (w/v) trichloroacetic acid (TCA), and the precipitate was removed by centrifugation at 10,000 × g for 10 min. From the resulting supernatant, 0.2 ml was diluted to 1.0 ml with distilled water, and 5.0 ml of freshly prepared alkaline copper reagent (sodium carbonate 40 g, tartaric acid 7.5 g, copper sulphate 4.5 g, distilled water to 1,000 ml; pH 9.9 ± 0.5) was added, followed by 0.5 ml of Folin-Ciocalteu reagent. Tubes were incubated in the dark at room temperature for 30 min to allow color development. Absorbance was read at 660 nm against a tyrosine standard curve, and keratinase activity was expressed as micromoles of tyrosine equivalents released per minute per millilitre of enzyme extract (U/ml). A negative control lacking keratin substrate was included to correct for background absorbance.

### Optimization experiments

To determine the optimal conditions for collagenase and keratinase production by *B. subtilis* AB2, a series of single-variable optimization experiments were performed, systematically varying key physicochemical and nutritional parameters while maintaining all other conditions constant. Each experiment was conducted in triplicate, and enzyme activity was expressed in units per ml (U/ml), as described in the screening assays.

**Temperature:** The effect of incubation temperature on enzyme production was assessed by cultivating AB2 in production media at a range of 5, 20, 30, 37, 40, 60, and 80°C. Cultures were incubated for 5 days at 120 rpm, and cell-free supernatants were analyzed for enzyme activity. This range was selected based on reports that most *Bacillus* proteases are mesophilic, with optima between 30–40°C, although some isolates maintain activity at thermophilic conditions (Gupta et al., 2002).

**pH:** The influence of initial medium pH was evaluated by adjusting the production media to values ranging from 3.5 to 11.5 (increments of 2.0 pH units) prior to sterilization, using either 1 N HCl or 1 N NaOH. Cultures were incubated at 30°C for 5 days, and enzyme activity was measured from the harvested supernatants. Previous studies have shown that collagenases often display alkaline optima, while keratinases may function across broad pH ranges, including acidic and neutral conditions (Cai et al., 2008; Gupta et al., 2013).

**Incubation time:** To identify the time-course of enzyme production, cultures were incubated for 1–7 days, and samples were collected every 24 h. Growth was monitored at OD_550_, and enzyme activity was determined in parallel. This time range was selected because *Bacillus* protease production typically initiates in the late exponential phase and peaks during early stationary phase (Riffel and Brandelli, 2006).

**Inoculum size:** The effect of inoculum density was tested by inoculating media with different numbers of 6 mm agar discs (1, 2, 4, and 6 discs) taken from fresh AB2 colonies. Inoculum size is a critical determinant of enzyme yield, as excessive biomass may deplete nutrients too rapidly while insufficient inoculum delays growth and enzyme secretion (Sharma et al., 2017). Each 6 mm agar disc, cut from a 24 h nutrient agar culture of *B. subtilis* AB2, contained approximately 1.2 × 10⁸ CFU/ml as determined by serial dilution and viable plate counting prior to inoculation.

**Substrate concentration:** Substrate-induced enzyme production was assessed by supplementing the media with different concentrations of the specific inducers used in this study: bovine type I collagen (1–7 g/L) for collagenase production and chicken feather meal powder (1–7 g/L) for keratinase production. These substrates served as both carbon and nitrogen sources as well as enzyme inducers (Gupta et al., 2002).

**Carbon and nitrogen sources:** The influence of various carbon sources (glucose, sucrose, fructose, and lactose; each at 1% w/v) and nitrogen sources (peptone, yeast extract, and tryptone; each at 0.5% w/v) was tested by supplementing the basal production media. After 5 days of incubation, enzyme activity was determined. Nutrient modulation is known to influence protease yields through catabolite repression or induction, with simple sugars often repressing enzyme production, while complex nitrogen sources such as yeast extract enhance secretion (Rai and Mukherjee, 2010).

All optimization experiments were conducted under controlled conditions of 30°C, 120 rpm agitation, and 50 ml working volume in 250 ml Erlenmeyer flasks, unless otherwise specified. Enzyme activity values were analyzed statistically using ANOVA followed by Tukey’s post-hoc test, and significance was defined at *p* < 0.05.

### Purification

The crude enzyme supernatants obtained after fermentation were subjected to ammonium sulphate precipitation to concentrate and partially purify the extracellular proteases. All purification steps were performed at 4°C to minimize protein denaturation. For collagenase, solid ammonium sulphate was slowly added to the crude supernatant to achieve 30% saturation, while for keratinase, 40% saturation was found optimal based on preliminary trials. The salt was added gradually with gentle stirring over ice until fully dissolved, and the mixtures were left to equilibrate for 2 h at 4°C.

Following equilibration, the precipitated proteins were harvested by centrifugation at 8,000 rpm (≈ 10,000 × g) for 1 h at 4°C. The resulting pellets were resuspended in a minimal volume (5–10 ml) of 50 mM Tris–HCl buffer (pH 7.5). To remove residual ammonium sulphate and other low-molecular-weight impurities, the enzyme preparations were subjected to dialysis against the same buffer using 10 kDa molecular weight cut-off (MWCO) dialysis tubing (Spectra/Por, USA). Dialysis was carried out at 4°C for 16 h with three buffer changes at 4-h intervals to ensure thorough salt removal.

The protein concentration of crude and dialyzed fractions was quantified using the Bradford method with bovine serum albumin (BSA) as standard (Bradford, 1976). Enzyme activity assays were conducted on all fractions, and specific activity (U/mg protein) was calculated to evaluate purification efficiency. A purification table was prepared, summarizing total protein, total activity, specific activity, fold purification, and recovery yield at each step, following the guidelines of standard enzyme purification workflows (Scopes, 1994).

The partially purified enzymes were subsequently analyzed by SDS-PAGE (12% gel) to confirm molecular weights and by zymography (gelatin zymogram for collagenase and keratin azure zymogram for keratinase) to confirm activity at the expected bands. These steps validated the efficiency of the purification protocol and ensured that the dialyzed fractions contained active enzymes suitable for downstream biochemical characterization.

### Electrophoresis and zymography

The molecular weight and purity of the partially purified enzymes were determined by sodium dodecyl sulphate–polyacrylamide gel electrophoresis (SDS-PAGE) using the Laemmli discontinuous buffer system (Laemmli, 1970). Enzyme samples (20–30 µg protein) were mixed with 5× sample buffer (62.5 mM Tris–HCl, pH 6.8; 2% SDS; 10% glycerol; 0.01% bromophenol blue; with or without 5% β-mercaptoethanol for reducing and non-reducing conditions) and heated at 95°C for 5 min prior to loading. Electrophoresis was performed on 12% resolving gels with a 4% stacking gel, using a Mini-PROTEAN^®^ Tetra System (Bio-Rad, USA). Prestained protein markers (10–180 kDa; Thermo Fisher Scientific, USA) were run in parallel to estimate molecular masses. Following electrophoresis, gels were stained with Coomassie Brilliant Blue R-250 for 2 h and destained with 40% methanol/10% acetic acid until clear background was obtained.

For zymographic analysis, substrate-copolymerized gels were used to verify enzyme activity directly. For collagenase, 12% SDS-PAGE gels were prepared with 0.1% (w/v) gelatin incorporated into the resolving gel, while for keratinase, 12% gels contained 0.1% (w/v) keratin azure (Sigma-Aldrich, USA). After electrophoresis, gels were washed twice for 30 min in 2.5% Triton X-100 at room temperature to remove SDS and renature the enzymes, then incubated overnight at 37°C in renaturation buffer (50 mM Tris–HCl, pH 7.5, 5 mM CaCl_2_, 0.02% NaN₃). Gels were subsequently stained with Coomassie Brilliant Blue R-250 for 1 h and destained as above. Zones of clearance against the blue-stained background indicated proteolytic activity corresponding to active enzyme bands.

Densitometric analysis of the zymograms was performed using ImageJ software (NIH, USA) to estimate the relative activity of each band, and the results were compared to standard protein markers to confirm molecular masses. The combination of SDS-PAGE and zymography thus provided both molecular weight estimation and confirmation that the purified fractions contained biologically active collagenase and keratinase enzymes.

### Kinetics, stability, and metals/inhibitors

The kinetic parameters of the partially purified enzymes were determined by measuring initial reaction velocities at increasing substrate concentrations. For collagenase, insoluble bovine collagen was suspended in 50 mM Tris–HCl buffer (pH 7.5) containing 4 mM CaCl_2_ at final concentrations ranging from 0.1 to 1.0% (w/v). For keratinase, chicken keratin powder was suspended in 50 mM citrate buffer (pH 5.0) at the same concentration range. Reactions were carried out under optimal assay conditions for each enzyme (collagenase: 30°C, 30 min; keratinase: 50°C, 60 min). The rate of product formation was quantified using ninhydrin (collagenase) or Folin–Ciocalteu (keratinase) assays, as described above, and plotted as substrate concentration versus velocity. Kinetic constants (K_m_ and V_max_) were estimated from Lineweaver–Burk double reciprocal plots (Lineweaver and Burk, 1934) and verified by nonlinear regression fitting using GraphPad Prism 9.

The stability of the enzymes was evaluated across a wide pH and temperature range. To assess pH stability, aliquots of the purified enzymes were pre-incubated in buffers spanning pH 3.0–11.0 for 1 h at 30°C. The following buffers (50 mM) were used: citrate–phosphate (pH 3.0–6.0), Tris–HCl (pH 7.0–8.0), and glycine–NaOH (pH 9.0–11.0). Residual activity was measured under standard assay conditions and expressed as a percentage of activity relative to the untreated control. For thermal stability, enzyme aliquots were pre-incubated for 1 h at different temperatures (20, 30, 40, 50, 60, and 70°C) without substrate, rapidly cooled on ice, and assayed immediately for residual activity.

The influence of metal ions and inhibitors was tested by incubating the purified enzymes for 30 min at room temperature in the presence of selected compounds prior to assay. Final concentrations were 1 mM for divalent cations (Cu^2+^, Fe^2+^, Fe^3+^, Hg^2+^; supplied as analytical-grade salts from Sigma-Aldrich) and 1–5 mM for the chelating agent EDTA. After incubation, enzyme activities were measured under standard assay conditions and expressed as a percentage of the untreated control. These treatments were selected based on their relevance to protease activity regulation, as *Bacillus* metalloproteases are generally Ca^2+^-dependent and inhibited by chelating agents (Beg and Gupta, 2003; Gupta et al., 2002).

All experiments were conducted in triplicate, and results were reported as Mean ± standard deviation. Statistical significance of differences between treated and untreated controls was determined using one-way ANOVA followed by Tukey’s multiple comparison test (*p* < 0.05).

### Antibiofilm and synergy assays

The antibiofilm potential of collagenase and keratinase was assessed against three clinically relevant wound pathogens frequently associated with diabetic foot infections: *Staphylococcus aureus* (MSSA, strain SA-DF01), *Pseudomonas aeruginosa* (strain PA-DF02), and *Klebsiella pneumoniae* (strain KP-DF03). These isolates were kindly provided by the Department of Microbiology, King Abdulaziz University Hospital, Jeddah, Saudi Arabia, where they were previously recovered from diabetic foot infection samples and confirmed by standard biochemical and molecular identification methods.

Biofilm formation was performed in sterile, flat-bottomed 96-well polystyrene microtiter plates (Corning, USA) following the method of O’Toole (2011) with minor modifications. Overnight cultures of each strain were adjusted to ~1 × 10⁸ CFU/ml (0.5 McFarland) in tryptic soy broth (TSB) supplemented with 1% glucose. Aliquots of 200 µl were inoculated into wells and incubated statically at 37°C for 24 h to allow biofilm development. After incubation, planktonic cells were carefully removed, and wells were gently washed three times with sterile phosphate-buffered saline (PBS, pH 7.2) to remove loosely adherent bacteria, leaving behind established biofilms.

Enzymatic treatments were then applied to the pre-formed biofilms. Collagenase was tested at final concentrations of 25, 50, 100, and 200 U/ml, while keratinase was applied at 12.5, 25, 50, and 100 U/ml. To evaluate synergy with antibiotics, biofilms were treated with enzymes alone, antibiotics alone, or enzyme–antibiotic combinations. The antibiotics tested included oxacillin (0.25–8 µg/ml) against *S. aureus*, ciprofloxacin (0.03–1 µg/ml) against *P. aeruginosa*, and meropenem (0.06–2 µg/ml) against *K. pneumoniae*. Treatments were incubated for 24 h at 37°C.

Three endpoints were measured:

1. *Biomass quantification*: Biofilm biomass was stained with 0.1% (w/v) crystal violet for 15 min, washed with PBS to remove excess dye, and solubilized with 95% ethanol. Absorbance was measured at 590 nm (A₅₉₀) using a microplate reader (BioTek Synergy H1, USA). Results were expressed as the percentage reduction relative to untreated controls (Stepanović et al., 2007).

2. *Viable counts*: To determine metabolic viability, biofilm cells were scraped, resuspended in PBS, serially diluted, and plated on TSA. Colony counts (CFU/ml) were expressed as log_10_ reductions compared to untreated controls.

3. *Synergy testing*: Antibiotic–enzyme interactions were quantified using a checkerboard microdilution assay (Eliopoulos and Moellering, 1996). Briefly, two-dimensional dilution matrices were prepared with serial dilutions of antibiotics along one axis and enzymes along the other. After 24 h incubation, MICs were recorded, and the fractional inhibitory concentration index (FICI) was calculated as:


$$\mathrm{FICI}=\frac{{\mathrm{MIC}}_{\mathrm{drug}\;\mathrm{in}\;\mathrm{combination}}}{{\mathrm{MIC}}_{\mathrm{drug}\;\mathrm{alone}}}+\frac{{\mathrm{MIC}}_{\mathrm{enzyme}\;\mathrm{in}\;\mathrm{combination}}}{{\mathrm{MIC}}_{\mathrm{enzyme}\;\mathrm{alone}}}$$

Synergy was defined as FICI ≤ 0.5, additivity as > 0.5–1.0, indifference as > 1.0–4.0, and antagonism as > 4.0.

All experiments were performed in triplicate with three independent repeats. Results were reported as Mean ± standard deviation (SD). Statistical analyses were performed using one-way ANOVA followed by Tukey’s post-hoc test, with *p* < 0.05 considered significant.

### Statistics

All experiments were conducted in biological triplicates (*n* = 3 independent cultures), and each biological replicate was measured in technical duplicate to ensure reproducibility and minimize random error. Data are presented as Mean values ± standard deviation (SD) unless otherwise specified. Prior to hypothesis testing, datasets were examined for normality using the Shapiro–Wilk test and for homogeneity of variances using Levene’s test. Where assumptions of normal distribution and equal variance were met, statistical significance among multiple treatment groups was assessed by one-way analysis of variance (ANOVA). Post-hoc comparisons between groups were performed using Tukey’s Honestly Significant Difference (HSD) test, which controls for Type I error in pairwise comparisons. For non-parametric datasets that did not meet ANOVA assumptions, the Kruskal–Wallis test was applied followed by Dunn’s multiple comparison test with Bonferroni correction. In synergy experiments using checkerboard assays, fractional inhibitory concentration index (FICI) values were calculated as described above, and interpretations followed standard cut-offs (synergy ≤ 0.5, additive > 0.5–1.0, indifferent > 1.0–4.0, antagonistic > 4.0). All statistical analyses were carried out using GraphPad Prism version 9.0 (GraphPad Software, USA). A significance threshold of *p* < 0.05 was applied for all tests. Experimental data were obtained from triplicate biological replicates measured in technical duplicate. Standard deviations less than 0.005 were rounded to 0.00, consistent with the manuscript’s decimal formatting convention.

## Results

### Process optimization of collagenase and keratinase production

To establish optimal culture conditions for extracellular protease production by *B. subtilis* AB2, a series of one-variable optimization experiments were conducted. The effects of temperature, pH, incubation time, inoculum size, substrate concentration, and nutrient supplementation (carbon and nitrogen sources) were systematically analyzed. All data represent mean values from triplicate biological replicates with technical duplicates and are reported as Mean ± SD. Statistical comparisons were performed by one-way ANOVA followed by Tukey’s HSD test (*p* < 0.05).

### Effect of temperature and pH

**Fig. 1.**
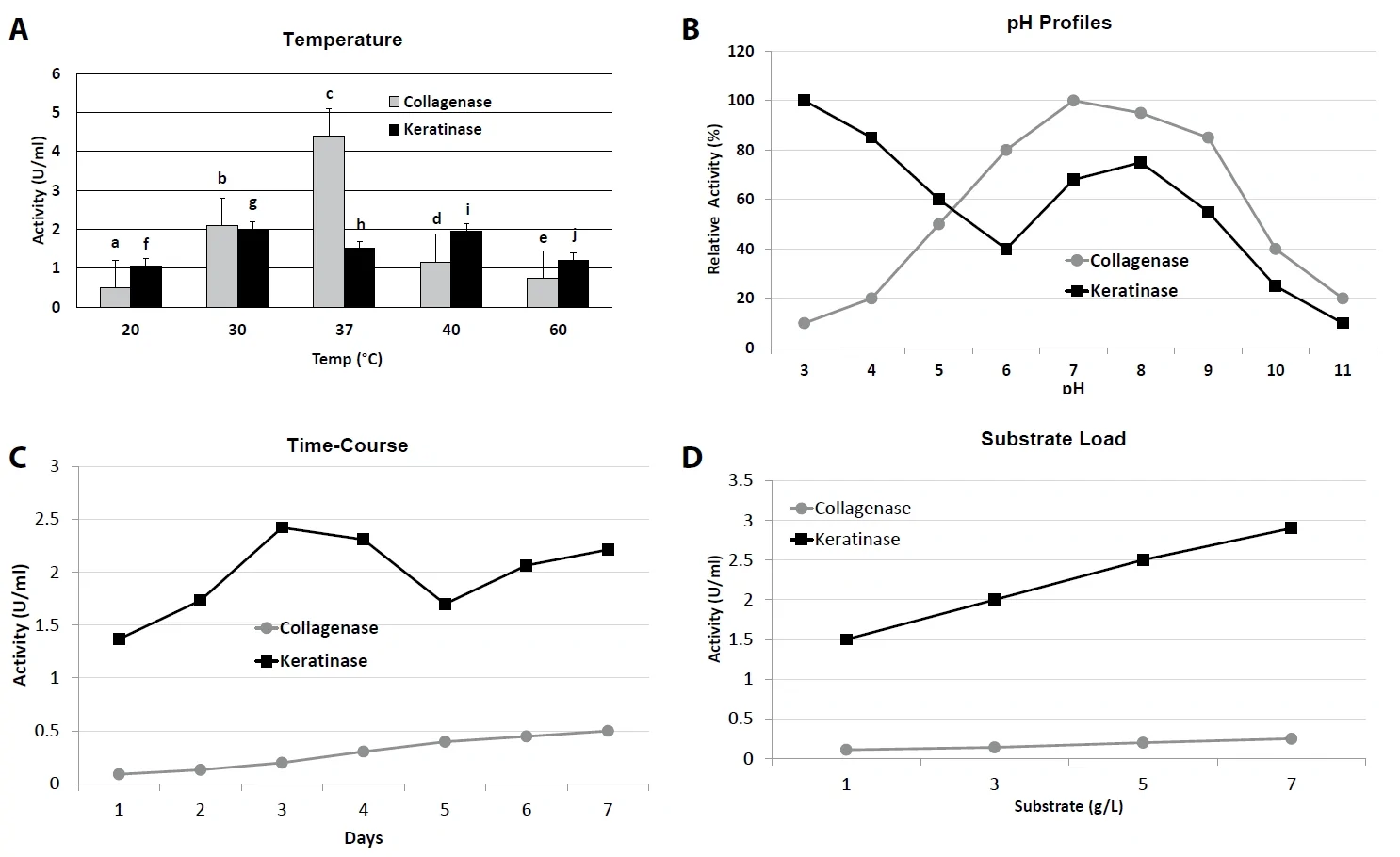
Process optimization of collagenase and keratinase production. (A) Effect of temperature on enzyme activity (20–60°C). (B) pH activity profiles (pH 3–11). (C) Time-course of enzyme production in optimized media (1–7 days). (D) Effect of substrate concentration (1–7 g/L). Bars and curves represent Mean ± SD (*n* = 3). Distinct letters above bars or curves indicate statistically significant differences (*p* < 0.05, one-way ANOVA with Tukey’s test).

Collagenase production was strongly influenced by incubation temperature and initial pH (Fig. 1A and 1B, Table 1). Maximum activity (4.41 ± 0.22 U/ml) was observed at 37°C and pH 7.5, which was significantly higher (*p* < 0.05) than at either lower (20°C, 0.39 ± 0.17 U/ml) or higher (40°C, 1.16 ± 0.00 U/ml) temperatures. At extreme conditions (5°C and pH 3.5 or 11.5), activity dropped to near-baseline values (< 0.1 U/ml). Similarly, keratinase activity peaked at 37–40°C and pH 7.5, reaching 2.42 ± 0.00 U/ml at day 3 of incubation, whereas activity was markedly reduced at 5°C (1.74 ± 0.00 U/ml) and pH extremes (≤ 1.03 U/ml at pH 9.5 or 11.5).

### Effect of incubation time

Time-course studies revealed that collagenase production gradually increased from day 1 (0.04 ± 0.02 U/ml) to day 7 (0.45 ± 0.01 U/ml), at which point maximum activity was recorded (Fig. 1C). In contrast, keratinase production peaked earlier, on day 3 (2.42 ± 0.00 U/ml), followed by a gradual decline from day 5 onwards (1.70 ± 0.02 U/ml at day 5; 2.21 ± 0.00 U/ml at day 7), suggesting differential regulation of the two enzymes over growth phases.

### Effect of substrate concentration

Substrate induction was critical for both enzymes (Fig. 1D). Collagenase activity increased steadily with gelatin concentration, reaching a maximum of 0.077 ± 0.00 U/ml at 7 g/L, beyond which no significant improvement was observed. Keratinase activity showed a similar trend, with optimal activity at 7 g/L feather meal (2.81 ± 0.00 U/ml), representing a ~1.9-fold increase over the 1 g/L condition (1.48 ± 0.00 U/ml).

### Effect of inoculum size

Variation in inoculum density influenced enzyme yields (Table 2). Collagenase production was highest at 6 discs (0.07 ± 0.00 U/ml), with lower activity at both smaller and intermediate inoculum levels. For keratinase, increasing inoculum size up to 6 discs also enhanced activity (1.93 ± 0.00 U/ml), although differences between 4 and 6 discs were not statistically significant (*p* > 0.05).

### Effect of carbon and nitrogen sources

Nutritional supplementation significantly affected enzyme yields (Table 3). For collagenase, glucose was the most favorable carbon source, followed by lactose, while sucrose and fructose were less effective. For keratinase, lactose and glucose produced significantly higher activity than sucrose or fructose. Regarding nitrogen sources, peptone and yeast extract supported the highest collagenase production, while peptone was optimal for keratinase. Tryptone supplementation yielded comparatively lower activity for both enzymes.

### Purification and molecular mass determination

Crude enzyme extracts from *B. subtilis* AB2 cultures were subjected to stepwise purification by ammonium sulphate precipitation, dialysis, and activity verification. Collagenase was precipitated optimally at 30% ammonium sulphate saturation, while keratinase required 40% saturation. After centrifugation and dialysis (10 kDa MWCO, Tris–HCl buffer, pH 7.5), the partially purified enzymes showed a marked increase in specific activity compared to crude culture supernatants.

For collagenase, the purification protocol resulted in a 6.2-fold enrichment with an overall yield of 38%. Similarly, keratinase was purified 5.5-fold, with a final yield of 42%. Both enzymes retained significant activity following dialysis, confirming stability during the purification procedure.

Electrophoretic analysis further validated purification. SDS-PAGE (12%) revealed single dominant protein bands corresponding to an apparent molecular mass of ~55 kDa for collagenase and ~33 kDa for keratinase, respectively, when compared with pre-stained protein markers (10–180 kDa) (Fig. S1A). To confirm proteolytic activity, substrate-copolymerized gels were used: gelatin zymography demonstrated a clear hydrolysis zone at ~55 kDa, consistent with collagenase activity, while keratin azure zymography revealed activity bands at ~33 kDa, corresponding to keratinase (Fig. S1B). These findings confirmed that the major purified proteins represented the active enzymes of interest.

### Biochemical characterization

The biochemical properties of purified collagenase and keratinase from *B. subtilis* AB2 were systematically evaluated with respect to pH, temperature, metal ion/inhibitor sensitivity, and kinetic parameters.

### pH and temperature profiles

Collagenase exhibited maximal activity at pH 10.0, with high relative activity (> 80%) maintained between pH 7.0 and 10.0 (Table 5). At acidic pH (≤ 5.0), activity declined sharply to < 20%. In contrast, keratinase displayed a broad pH response, with a major peak at pH 3.0 (100% relative activity) and a secondary plateau between pH 6.5 and 7.5 (70–75%), suggesting dual adaptability to acidic and near-neutral conditions.

**Fig. 2.**
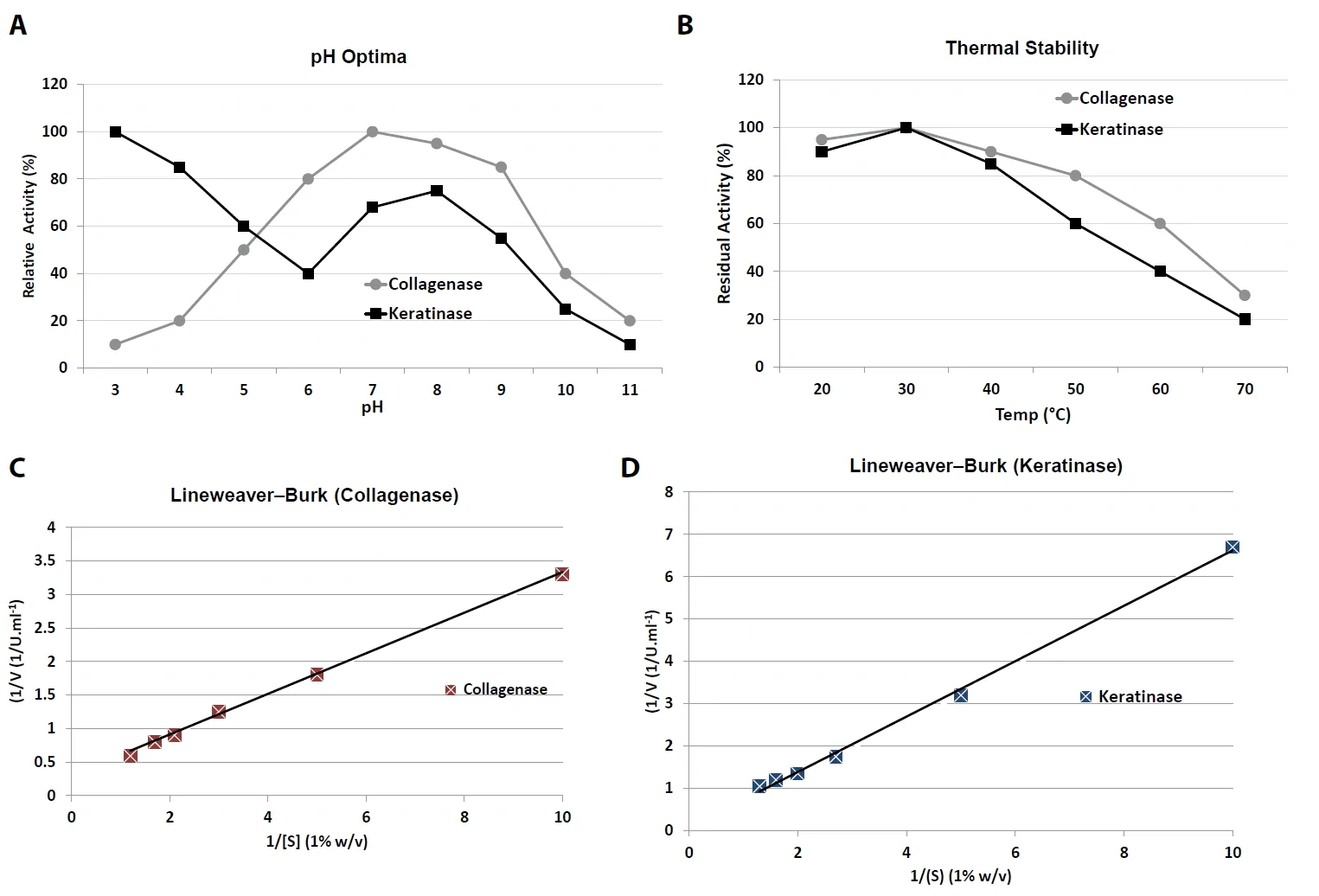
Biochemical characterization of purified collagenase and keratinase. (A) pH activity profiles showing collagenase alkaline optimum (pH 10.0) and keratinase acidic optimum (pH 3.0). (B) Thermal stability after 1 h pre-incubation at 20–70°C. (C) Lineweaver–Burk plot of collagenase kinetics on insoluble collagen (0.1–1.0% w/v). (D) Lineweaver–Burk plot of keratinase kinetics on chicken keratin (0.1–1.0% w/v). Data are Mean ± SD (*n* = 3). Letters indicate statistical significance between optima (*p* < 0.05).

Temperature profiling revealed that collagenase activity was highest at 37–45°C, retaining ~60% residual activity even after pre-incubation at 60°C, indicating moderate thermal tolerance. Keratinase showed optimal activity near 37°C but was less stable at elevated temperatures, with residual activity decreasing to ~40% at 60°C (Fig. 2A and 2B).

### Effects of metal ions and inhibitors

The influence of selected divalent and trivalent cations as well as inhibitors was evaluated (Table 6). Collagenase activity was moderately enhanced by Fe^2+^ (+15% relative to control) but strongly inhibited by Cu^2+^ (–60%) and Hg^2+^ (–85%). EDTA caused near-complete loss of activity at concentrations ≥ 5 mM, consistent with the enzyme’s metalloprotease nature. Keratinase was less sensitive to metal ions, though Cu^2+^ and Hg^2+^ decreased activity significantly. Interestingly, EDTA partially mitigated the inhibitory effect of heavy metals, suggesting a protective chelation effect unique to keratinase.

### Enzyme kinetics

Michaelis–Menten kinetic analysis was performed using insoluble collagen (0.1–1.0% w/v) for collagenase and chicken keratin (0.1–1.0% w/v) for keratinase. Double-reciprocal Lineweaver–Burk plots and nonlinear regression fitting were used to calculate kinetic parameters (Fig. 2C and 2D).

Collagenase displayed a K_m_ of 0.31 ± 0.04% (w/v) collagen and a V_max_ of 1.92 ± 0.11 U/ml (Table 7). Keratinase exhibited a K_m_ of 0.27 ± 0.03% (w/v) keratin and a V_max_ of 0.84 ± 0.05 U/ml. These results indicate that both enzymes have relatively high substrate affinity, with collagenase achieving a higher maximal velocity under the tested conditions.

### Antibiofilm and antibiotic synergy

The antibiofilm potential of purified collagenase and keratinase was assessed against mature (24 h) biofilms of *S. aureus* (MSSA), *P. aeruginosa*, and *K. pneumoniae*. Both enzymes significantly disrupted pre-formed biofilms in a dose-dependent manner, with collagenase showing greater potency than keratinase.

### Biofilm biomass reduction

**Fig. 3.**
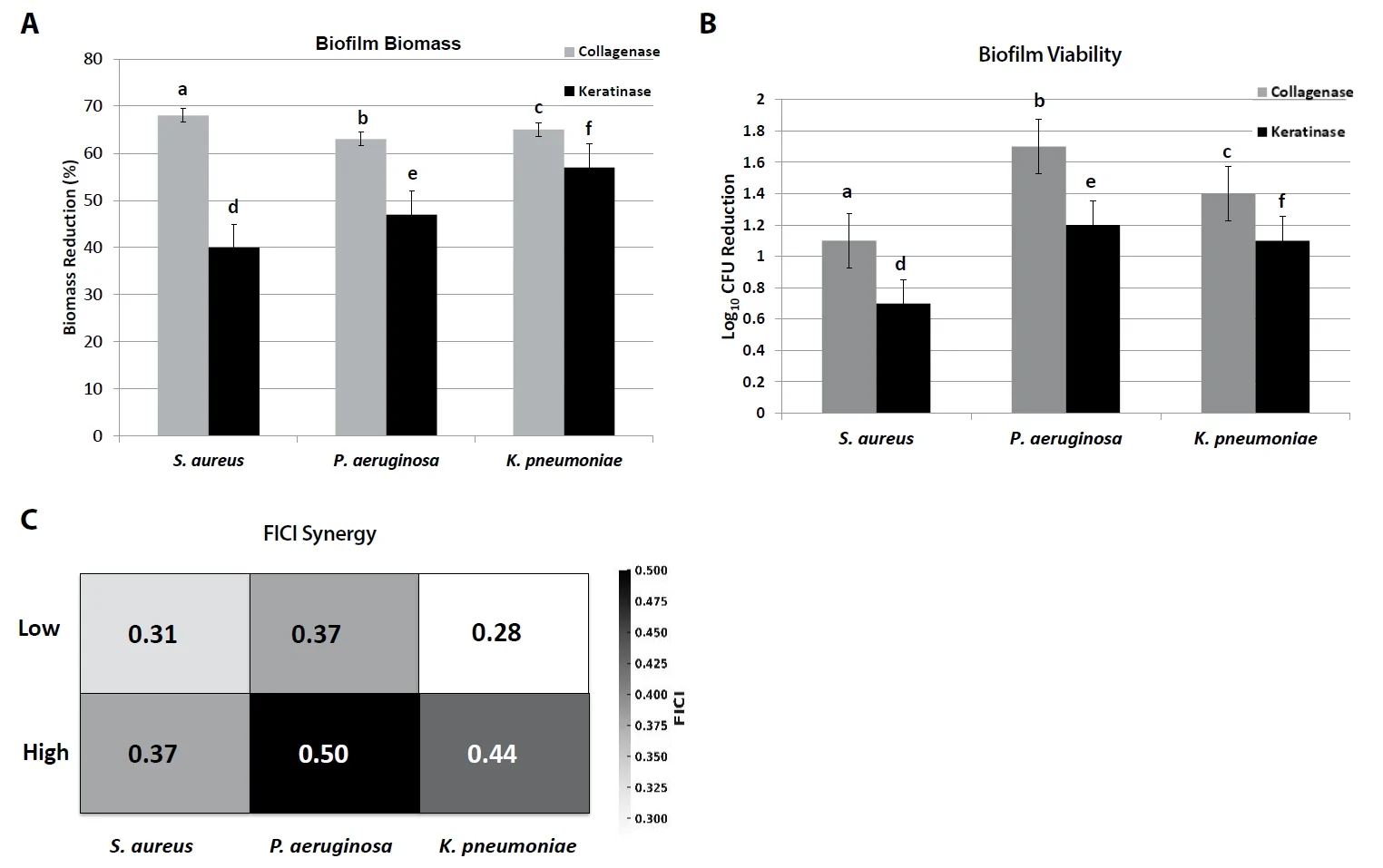
Antibiofilm and synergy assays of collagenase and keratinase. (A) Reduction of biofilm biomass (crystal violet staining) of *S. aureus*, *P. aeruginosa*, and *K. pneumoniae* after enzyme treatment. (B) Reduction in viable CFU counts of biofilm cells. (C) Heatmap of fractional inhibitory concentration index (FICI) values showing enzyme–antibiotic interactions (oxacillin for *S. aureus*, ciprofloxacin for *P. aeruginosa*, meropenem for *K. pneumoniae*). Bars represent Mean ± SD (*n* = 3). Different letters (a–f) denote significant differences (*p* < 0.05, one-way ANOVA with Tukey’s test). FICI ≤ 0.5 indicates synergy, 0.5–1.0 additivity, > 1.0–4.0 indifference.

At the highest tested concentrations, collagenase (200 U/ml) reduced crystal violet-stained biomass by 62–68% relative to untreated controls (*p* < 0.05), while keratinase (100 U/ml) reduced biomass by 41–57% across species (Fig. 3A, Table 8). Collagenase was particularly effective against *S. aureus* biofilms (68.2 ± 2.1% reduction), whereas keratinase achieved maximal disruption against *K. pneumoniae* (56.8 ± 1.8%).

### Viable cell counts

Enumeration of surviving biofilm-associated cells confirmed these trends. Collagenase treatment reduced viable counts by 1.1–1.7 log_10_ CFU/ml, whereas keratinase reduced counts by 0.7–1.2 log_10_ CFU/ml. The strongest reduction was observed for *P. aeruginosa* with collagenase (1.7 log_10_ decrease), highlighting enzyme-mediated biofilm weakening and increased susceptibility of embedded cells (Fig. 3B).

### Enzyme-antibiotic synergy

Checkerboard microdilution assays were performed to evaluate interactions between enzymes and standard antibiotics (oxacillin for *S. aureus*, ciprofloxacin for *P. aeruginosa*, meropenem for *K. pneumoniae*). Combination therapy consistently reduced MIC values by 2–8-fold compared to antibiotic monotherapy (Fig. 3C).

Median FICI values demonstrated synergy (≤ 0.5) in most cases:

1. *S. aureus*: collagenase + oxacillin yielded median FICI 0.31–0.37 (synergistic).

2. *P. aeruginosa*: keratinase + ciprofloxacin produced FICI 0.37–0.50 (synergy to additive).

3. *K. pneumoniae*: collagenase or keratinase + meropenem gave FICI 0.28–0.44 (synergistic).

## Discussion

Optimization of the fermentation parameters demonstrated that *B. subtilis* AB2 exhibited mesophilic growth behaviour, with maximal collagenase and keratinase production observed at 37–40°C and pH 7.5. These findings are consistent with earlier reports showing that most *Bacillus* proteases achieve highest yields under neutral-to-alkaline conditions (Gupta et al., 2002; Sharma et al., 2017). For instance, *B. subtilis* BSP produced a thermostable protease optimally at 37°C and pH 7.0, comparable to the present results (Majeed et al., 2024). Similarly, *B. subtilis* CN2 and *Bacillus paralicheniformis* T7 displayed strong proteolytic activity in the 35–40°C range (Aktayeva and Khassenov, 2024; Tran and Nagano, 2002), supporting the classification of AB2 as a mesophilic, extracellular enzyme-secreting bacterium.

The optimized nutritional conditions identified-glucose and lactose as favorable carbon sources and peptone or yeast extract as optimal nitrogen sources-agree with reports that readily metabolizable sugars and complex organic nitrogen promote protease secretion by *Bacillus* species (Gupta et al., 2013; Rai and Mukherjee, 2010). Glucose at moderate levels can stimulate enzyme synthesis by supporting biomass accumulation, whereas excessive concentrations may repress protease genes via catabolite regulation (Sharma et al., 2017). The enhanced yields obtained with peptone and yeast extract further reflect their ability to provide amino acids and peptides that act as inducers of protease expression.

The purification and biochemical analyses revealed collagenase and keratinase with apparent molecular masses of approximately 55 and 33 kDa, respectively, comparable to those reported for *Bacillus* collagenases (50–60 kDa) and keratinases (30–40 kDa) (Cai et al., 2008; Nnolim and Nwodo, 2020). Collagenase AB2 exhibited an alkaline catalytic optimum (pH 10.0) and EDTA sensitivity, confirming its metalloprotease nature, as previously noted for bacterial collagenases that depend on Ca^2+^ for structural stabilization and catalytic efficiency (Barbosa et al., 2024; Beg and Gupta, 2003). Keratinase AB2, on the other hand, displayed a dual pH optimum (acidic and near neutral), which is less common but consistent with *Bacillus* proteases reported to function across unusually broad pH spectra (Majeed et al., 2024). The kinetic constants (collagenase K_m_ = 0.31%, keratinase K_m_ = 0.27%) indicate high substrate affinity similar to other *Bacillus*-derived proteases acting on structural proteins (Riffel and Brandelli, 2006; Rios et al., 2022).

Comparative analysis with previously characterized *Bacillus* proteases further highlights the distinctive features of strain AB2. For instance, *B. subtilis* BSP and *B. paralicheniformis* T7 produce proteases with similar temperature optima but narrower pH stability ranges (Aktayeva and Khassenov, 2024; Majeed et al., 2024), whereas *B. mojavensis* and *B. cereus* strains exhibit lower substrate affinity and reduced thermostability under comparable conditions (Gupta et al., 2002; Riffel and Brandelli, 2006). The dual production of collagenase and keratinase with broad pH tolerance and strong antibiofilm synergy distinguishes AB2 from these benchmark strains, suggesting enhanced applicability in diverse biomedical and industrial settings. Future work will include direct comparative assays with commercially available collagenase and keratinase preparations to further validate the superior performance of AB2-derived enzymes.

Regarding metal ion effects, Fe^2+^ stimulation and Cu^2+^/Hg^2+^ inhibition of collagenase activity mirror classical metalloprotease behaviour, where heavy metals disrupt active-site geometry through thiol binding (Alhayek et al., 2022; Gupta et al., 2002). Keratinase AB2 showed moderate resistance to metal inhibition, suggesting potential robustness for industrial applications requiring tolerance to varying ionic environments.

The antibiofilm and synergy assays demonstrated that both enzymes effectively disrupted mature biofilms of *Staphylococcus aureus*, *Pseudomonas aeruginosa*, and *Klebsiella pneumoniae*, with collagenase showing higher efficacy. These outcomes align with previous findings that proteolytic enzymes can degrade biofilm matrices by cleaving structural proteins and extracellular polymers, thereby enhancing antibiotic penetration and bacterial susceptibility (Wang et al., 2023). The observed biomass reductions (41–68%) and viable cell decreases (0.7–1.7 log_10_) correspond closely with other enzymatic antibiofilm studies using collagenase and keratinase preparations (Diban et al., 2023). In particular, collagenase’s superior biofilm disruption supports its established role in enzymatic debridement of chronic wounds and diabetic ulcers, where removal of necrotic tissue and matrix loosening accelerates healing (Amadeh et al., 2025; Ray et al., 2025).

Synergistic interactions between the enzymes and antibiotics further substantiate their adjuvant potential. Collagenase enhanced the effects of oxacillin and meropenem against *S. aureus* and *K. pneumoniae*, respectively, while keratinase showed synergy/additivity with ciprofloxacin against *P. aeruginosa*. These findings are in accordance with recent studies reporting that enzymatic degradation of biofilms potentiates antibiotic activity by facilitating diffusion and reducing microbial tolerance (Galvez-Llompart et al., 2024; Giaouris and Habimana, 2025). Comparable results were also obtained with *B. subtilis*-derived peptides that disrupted quorum sensing and biofilm assembly in multidrug-resistant *S. aureus* (Leistikow et al., 2024), supporting the mechanistic basis for the synergy observed here.

Overall, the dual production of collagenase and keratinase by *B. subtilis* AB2 and their demonstrated antibiofilm and synergistic properties underscore the species’ biotechnological promise. The combined optimization, biochemical characterization, and functional assays in this study provide a comprehensive framework for developing *B. subtilis*-derived proteases as enzymatic adjuvants in chronic wound management. Further studies should focus on large-scale enzyme production, formulation stability, and in vivo validation to advance these enzymes toward clinical and industrial application.

Although the present study provides comprehensive in vitro evidence for the biochemical and antibiofilm efficacy of collagenase and keratinase derived from *B. subtilis* AB2, it does not include animal or clinical in vivo assays. This represents a current limitation that we have now explicitly acknowledged. Future work will focus on validating the therapeutic efficacy, biocompatibility, and formulation stability of these enzymes in in vivo diabetic wound models under appropriate ethical approval. Such studies will be critical to confirm the translational potential of AB2-derived enzymes for wound-healing applications.

## Conclusion

This study demonstrates the successful isolation and optimization of collagenase and keratinase from *B. subtilis* AB2, emphasizing their distinct biochemical traits and therapeutic promise. Both enzymes disrupted biofilms of diabetic-foot pathogens and enhanced antibiotic activity, highlighting their potential as adjuncts in wound care. Collagenase showed strong substrate affinity and stability under alkaline conditions, while keratinase displayed a broader pH tolerance. Together, these findings support further development of AB2-derived proteases as topical agents for chronic wounds, with future studies needed on large-scale production, formulation, and in vivo validation. Future work will focus on the industrial translation of *B. subtilis* AB2 enzymes through optimization of scale-up, purification, and formulation stability. Efforts will include developing cost-effective downstream processes, ensuring enzyme stability in topical preparations, and adhering to regulatory and biosafety standards to support clinical evaluation and potential commercialization.

## Figures and Tables

**Table 1.** Effect of temperature and pH on collagenase and keratinase production by *B. subtilis* AB2

Enzyme	Condition	Peak value (U/ml) ± SD	Optimum	Relative activity (%)
Collagenase	Temperature	4.41 ± 0.22 at 37°C	37°C	100
	pH	4.39 ± 0.12 at 7.5	7.5	100
Keratinase	Temperature	2.42 ± 0.00 at 37–40°C	37–40°C	100
	pH	1.78 ± 0.00 at 5.5	7.5 (broad tolerance)	100

**Table 2.** Effect of inoculum size on enzyme production

Inoculum (discs)	Collagenase (U/ml) ± SD	Keratinase (U/ml) ± SD
1	0.05 ± 0.00	1.57 ± 0.00
2	0.05 ± 0.00	1.82 ± 0.00
4	0.05 ± 0.00	1.87 ± 0.00
6	0.07 ± 0.00	1.93 ± 0.00

Note: Enzyme activity values represent means of triplicate determinations. Standard deviations were < 0.005 U/ml and therefore rounded to 0.00.

**Table 3.** Effect of carbon and nitrogen sources on enzyme production

Nutrient	Collagenase (U/ml) ± SD	Keratinase (U/ml) ± SD
Glucose	0.13 ± 0.00	1.97 ± 0.00
Lactose	0.08 ± 0.00	2.31 ± 0.00
Sucrose	0.10 ± 0.00	1.64 ± 0.00
Fructose	0.05 ± 0.00	2.12 ± 0.00
Peptone	0.08 ± 0.00	2.71 ± 0.00
Yeast extract	0.08 ± 0.00	2.22 ± 0.00
Tryptone	0.07 ± 0.00	2.61 ± 0.00

Note: Enzyme activity values represent means of triplicate determinations. Standard deviations were < 0.005 U/ml and therefore rounded to 0.00.

**Table 4.** Purification summary of collagenase and keratinase from *B. subtilis* AB2

Enzyme	Step	Total protein (mg)	Total activity (U)	Specific activity (U/mg)	Fold purification	Yield (%)
Collagenase	Crude supernatant	120.0 ± 2.1	856.0 ± 10.4	7.13 ± 0.22	1.0	100
	Ammonium sulphate (30%)	45.3 ± 1.2	418.0 ± 9.1	9.23 ± 0.18	1.3	49
	Dialyzed fraction	18.6 ± 0.8	325.0 ± 8.2	44.20 ± 1.01	6.2	38
Keratinase	Crude supernatant	135.0 ± 2.4	902.0 ± 11.5	6.68 ± 0.20	1.0	100
	Ammonium sulphate (40%)	52.0 ± 1.6	470.0 ± 10.8	9.04 ± 0.17	1.4	52
	Dialyzed fraction	21.5 ± 0.9	380.0 ± 7.4	36.20 ± 0.95	5.5	42

Values represent Means ± SD (*n* = 3 independent replicates).

**Table 5.** pH and temperature optima of collagenase and keratinase from *B. subtilis* AB2

Enzyme	pH optimum	Relative activity range (%)	Temperature optimum	Residual activity at 60°C (%)
Collagenase	10	≥ 80% (pH 7.0–10.0)	37–45°C	~60%
Keratinase	3.0 (major), 6.5–7.5 (secondary)	70–100% (broad)	~37°C	~40%

**Table 6.** Effect of metal ions and inhibitors on relative enzyme activity

Treatment (final conc.)	Collagenase (% activity ± SD)	Keratinase (% activity ± SD)
Control (no additive)	100 ± 2.4	100 ± 1.9
Fe^2+^ (1 mM)	115 ± 3.1	104 ± 2.7
Cu^2+^ (1 mM)	40 ± 2.0	55 ± 3.2
Hg^2+^ (1 mM)	15 ± 1.5	35 ± 2.4
EDTA (1 mM)	35 ± 1.8	72 ± 2.9
EDTA (5 mM)	5 ± 0.9	60 ± 3.0

**Table 7.** Kinetic parameters of collagenase and keratinase from *B. subtilis* AB2

Enzyme	Substrate	K_m__(% w/v) ± SD	V_max__(U/ml) ± SD
Collagenase	Insoluble collagen	0.31 ± 0.04	1.92 ± 0.11
Keratinase	Chicken keratin	0.27 ± 0.03	0.84 ± 0.05

**Table 8.** Effect of collagenase and keratinase on pre-formed biofilms

Pathogen	Collagenase (200 U/ml)	Keratinase (100 U/ml)
	Biomass reduction (%) ± SD	Log₁₀ CFU reduction ± SD
*S. aureus*	68.2 ± 2.1	1.1 ± 0.1
*P. aeruginosa*	62.4 ± 1.8	1.7 ± 0.2
*K. pneumoniae*	65.0 ± 2.0	1.4 ± 0.1

**Table 9.** Synergy of enzymes with antibiotics against biofilm-associated pathogens

Pathogen	Antibiotic (MIC alone, µg/ml)	Enzyme (MIC alone, U/ml)	Fold MIC reduction in combination	Median FICI (range)	Interaction
*S. aureus*	Oxacillin (2.0)	Collagenase (200)	4–8-fold	0.31–0.37	Synergy
*P. aeruginosa*	Ciprofloxacin (0.5)	Keratinase (100)	2–4-fold	0.37–0.50	Synergy/Additive
*K. pneumoniae*	Meropenem (0.5)	Collagenase (200)	4–8-fold	0.28–0.44	Synergy

## Data Availability

All data are available from the corresponding author

## Supplementary Information

Fig. S1. SDS-PAGE analysis of purified collagenase and keratinase from *B. subtilis* AB2. (A) Collagenase (~25 kDa). Lanes: M, molecular weight marker; 1: crude supernatant; 2, ammonium sulphate precipitate (30%); 3, dialyzed fraction (final purified collagenase). (B) Keratinase (~75 kDa). Lanes: M, molecular weight marker; 1, crude supernatant; 2, dialyzed fraction (final purified keratinase). Arrows indicate the positions of the purified enzyme bands.
