# Supplementary material for: Proteolytic enzymes from Bacillus subtilis AB2 as antibiofilm adjuvants: Bioprocess optimization, mechanistic insights, and synergy with antibiotics
Source: J Microbiol. 2025 Dec 31;63(12):e2509019. doi: 10.71150/jm.2509019 (PMC13577040; doi:10.71150/jm.2509019)
Supplement: Supplementary file 1 [file jm-2509019-Supplementary-Fig-S1.pdf]

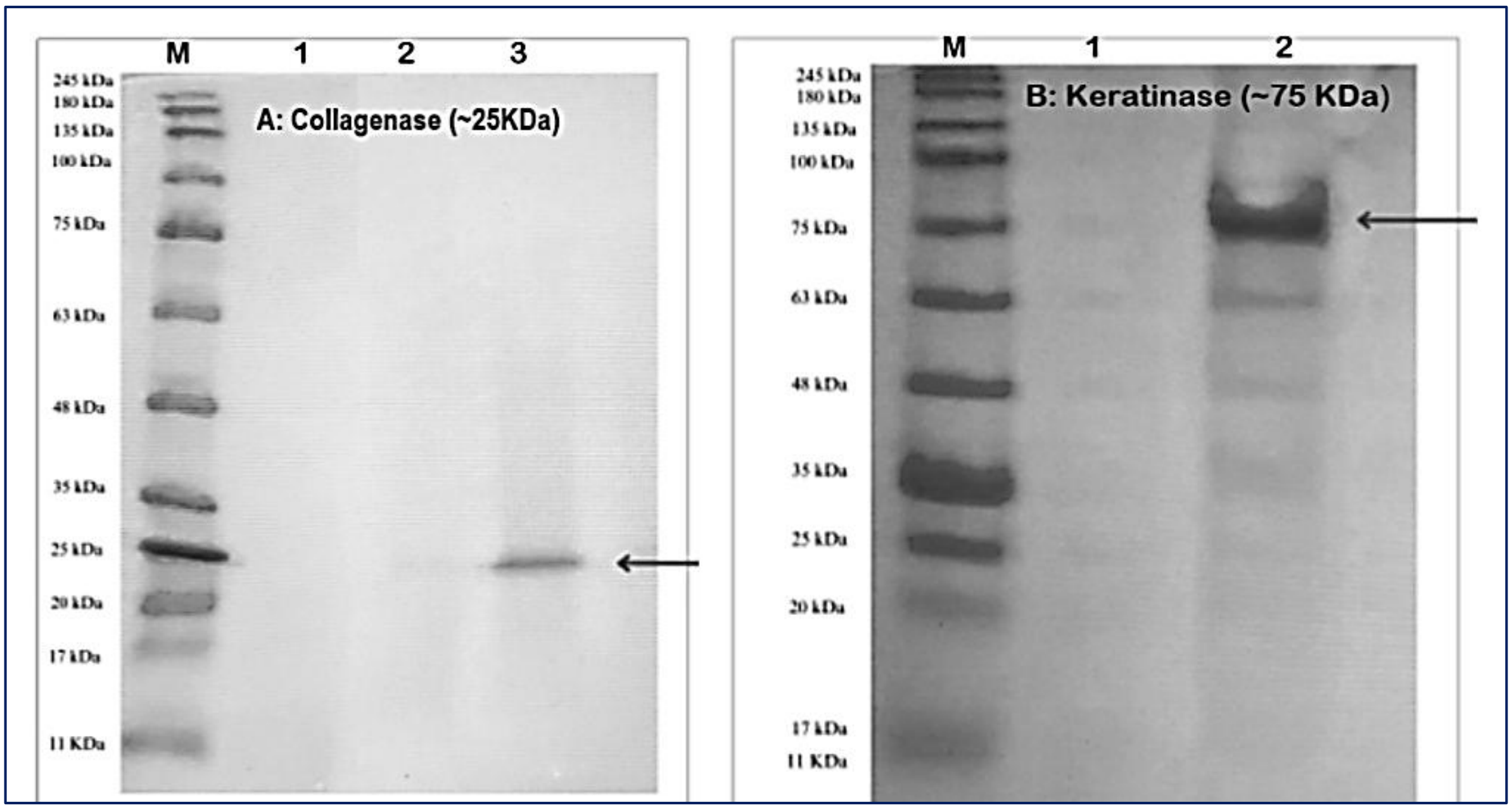

Fig. S1. SDS-PAGE analysis of purified collagenase and keratinase from *B. subtilis* AB2. (A) Collagenase (~25 kDa). Lanes: M, molecular weight marker; 1: crude supernatant; 2, ammonium sulphate precipitate (30%); 3, dialyzed fraction (final purified collagenase). (B) Keratinase (~75 kDa). Lanes: M, molecular weight marker; 1, crude supernatant; 2, dialyzed fraction (final purified keratinase). Arrows indicate the positions of the purified enzyme bands.
